# Type 2 Diabetes Related Mitochondrial Defects in Peripheral Mononucleated Blood Cells from Overweight Postmenopausal Women

**DOI:** 10.3390/biomedicines11010121

**Published:** 2023-01-03

**Authors:** Elisa Calabria, Valentina Muollo, Valentina Cavedon, Teodora Capovin, Leonardo Saccenti, Francesco Passarotti, Laura Ghiotto, Chiara Milanese, Matteo Gelati, Doriana Rudi, Gian Luca Salvagno, Giuseppe Lippi, Enrico Tam, Federico Schena, Silvia Pogliaghi

**Affiliations:** 1 Department of Neuroscience, Biomedicine and Movement Science, University of Verona, 37131 Verona, Italy; 2 Department of Medicine, University of Verona, 37131 Verona, Italy; 3 CeRiSM-Centro Sport Montagna e Salute, 38068 Rovereto, Italy

**Keywords:** mitochondria, type 2 diabetes, Hb1Ac, biomarker, PBMC

## Abstract

Type 2 diabetes (T2D) is a multisystem disease that is the subject of many studies, but the earliest cause of the disease has yet to be elucidated. Mitochondrial impairment has been associated with diabetes in several tissues. To extend the association between T2D and mitochondrial impairment to blood cells, we investigated T2D-related changes in peripheral mononucleated blood cells’ (PBMCs) mitochondrial function in two groups of women (CTRL vs. T2D; mean age: 54.1 ± 3.8 vs. 60.9 ± 4.8; mean BMI 25.6 ± 5.2 vs. 30.0 ± 5), together with a panel of blood biomarkers, anthropometric measurements and physiological parameters (VO_2max_ and strength tests). Dual-energy X-ray absorptiometry (DXA) scan analysis, cardio-pulmonary exercise test and blood biomarkers confirmed hallmarks of diabetes in the T2D group. Mitochondrial function assays performed with high resolution respirometry highlighted a significant reduction of mitochondrial respiration in the ADP-stimulated state (OXPHOS; −30%, *p =* 0.006) and maximal non-coupled respiration (ET; −30%, *p =* 0.004) in PBMCs samples from the T2D group. The total glutathione antioxidant pool (GSHt) was significantly reduced (−38%: *p =* 0.04) in plasma samples from the T2D group. The fraction of glycated hemoglobin (Hb1Ac) was positively associated with markers of inflammation (C-reactive protein-CRP r = 0.618; *p =* 0.006) and of dyslipidemia (triglycerides-TG r = 0.815; *p* < 0.0001). The same marker (Hb1Ac) was negatively associated with mitochondrial activity levels (OXPHOS r = −0.502; *p =* 0.034; ET r = −0.529; *p =* 0.024). The results obtained in overweight postmenopausal women from analysis of PBMCs mitochondrial respiration and their association with anthropometric and physiological parameters indicate that PBMC could represent a reliable model for studying T2D-related metabolic impairment and could be useful for testing the effectiveness of interventions targeting mitochondria.

## 1. Introduction

Type 2 diabetes (T2D) is a worldwide epidemic disease, and its prevalence has a large and growing spread, not only in Western countries, but also in “emerging” economies, such as China and India [1]. In recent years, there has been a growing interest in understanding whether mitochondria are involved in the onset of the disease and whether mitochondrial function parameters can be used as biomarkers of the disease. Certainly, mitochondrial defects have been associated with T2D both in terms of reduction of the expression of genes involved in mitochondrial biogenesis and of mitochondrial abundance, dynamics, and function [2].

The diagnosis of diabetes and pre-diabetes relies on indexes of glycemic control, e.g., conventional fasting plasma glucose (FPG), impaired glucose tolerance (IGT) or glycated hemoglobin (Hb1Ac). While the first two are indexes of acute dysglycemia, Hb1Ac is a marker of chronic dysglycemia [3]. Almost half of the adult population aged 65 and above have a pre-diabetic condition, and the distribution of increased Hb1Ac is much higher in women than in men [4]. The literature is richer in studies on diabetic patients than on pre-diabetic subjects, but it is becoming evident that there is a relation between the continual increase in Hb1Ac and a related greater risk of diabetes, and that in the early steps of this increase, the condition can still be reversed with lifestyle interventions [3,5]. 

The primary causes for the development of T2D are typically set in obesity and in physical inactivity, or in an impaired balance between metabolic load versus metabolic capacity [6]. According to this concept, in relation to diabetes, metabolic load components are represented by dietary glycemic load, adiposity (mainly abdominal adiposity), sedentary lifestyle and stress. On the other hand, metabolic capacity is represented by pancreatic function, through insulin secretion, and muscle mass, a key player in glucose clearance [6]. T2D is usually accompanied by several comorbidities such as dyslipidemia, cardiomyopathies, and microvascular complications that could be partially prevented with early interventions. 

T2D-related dyslipidemia is characterized by elevated triglycerides (TG), elevated low-density lipoprotein (LDL), and decreased levels of high-density lipoprotein (HDL) [7,8], and the TG/HDL ratio is also positively associated with T2D and its onset [9,10,11]. 

In adults with T2D, the aerobic cardio-respiratory capacity is further reduced compared to healthy subjects, even in the absence of overt cardiovascular disease [12,13]. This is relevant since the maximal aerobic capacity (VO_2max_) is a predictor of risk for the development of cardiovascular disease, heart failure and mortality [14,15]. A poor VO_2max_ is further associated with an insufficient level of physical activity, and this factor may have a pivotal role in the development of T2D. It is well known that individuals with a physically active lifestyle have a lower risk of developing T2D compared to sedentary people [16,17]. On the other hand, an increase in physical activity reaching the minimal recommended guidelines (i.e., 150 min of moderate intensity per week) would be associated with a 26% lower risk of T2D [17]. 

Although the reasons are still unknown, the risk of diabetes, its side effects and the outcome of treatment have sex-specific differences. The risk of diabetes in people with overweight or obesity is higher in women than in men (eight- and six-fold, respectively) [18,19]. Women are also more susceptible to the deleterious side effects of T2D and hypertension on cardiovascular health, and the protective effect of therapeutic interventions is more effective for men than for women [20,21,22,23]. In light of these considerations, we chose to investigate overweight postmenopausal women, since in this population, T2D is the most common chronic disease [24].

Mitochondria, which are involved in aging and obesity, have been implicated in insulin resistance in T2D [25]. In diabetes, a reduction of the cellular capacity to efficiently respond to insulin stimulation, together with impaired insulin secretion and action, may explain many alterations of the mitochondrial function [26]. However, the precise molecular mechanisms leading to mitochondrial impairment and the cause–effect relationships linking mitochondrial dysfunction to the disease have not yet been clarified. Mitochondrial dysfunction has a greater impact in tissues with higher mitochondrial content (i.e., skeletal muscle, liver, brain, and heart) [27,28]. Many studies have demonstrated T2D-related alterations of mitochondria both in terms of gene expression and enzyme content and activity [29,30,31]. At the molecular level, analysis of gene arrays associated T2D with decreased expression of genes involved in mitochondrial maintenance, such as *PGC1A*, *NRF1* and several key players of mitochondrial function [29,32].

Decreased mitochondrial enzymatic activity, content, and fatty acids oxidation have been reported in muscles from adults with obesity and/or T2D, as well as in T2D animal experimental models. [30]. In samples from the vastus lateralis muscle of diabetic patients, there is a decreased mitochondrial respiratory capacity, in particular the phosphorylative capacity stimulated by ADP and sustained by complex I (with pyruvate and malate substrates) [30]. Reduced mitochondrial activity, namely decreased oxidative phosphorylation (OXPHOS) at the level of complex I and III, has also been reported in a diabetic mouse model [33]. Although skeletal muscle is the most frequently used tissue to study the mechanisms related to T2D, insulin resistance (IR) can also impact other tissues such as the liver, adipose tissue, brain, cardiac muscle and the gastro-intestinal system [27,34]. In each tissue, the phenotype associated with T2D is rather specific, but the underlying molecular pathways impaired are often common [34,35].

In this study, we used peripheral mononucleated blood cells (PBMCs) to investigate mitochondrial activity in relation to T2D in overweight post-menopausal women. An increasing number of studies indicate that PBMCs can reflect mitochondrial function at the systemic level [36,37]. In recent years, both platelets (PLTs) and PBMCs have been proposed as surrogates for skeletal muscle biopsies to investigate the mitochondrial phenotype, and it has been proposed that circulating cells may inform on mitochondrial health or dysfunction at a systemic level [38]. Platelets are a very abundant and rather homogeneous cell population. However they are anucleate, and they have the possibility to degranulate and activate, hence requiring greater attention during the sampling and purification phases [39]. To these factors is added a very short turnover (every 8 days), making them suitable for punctual and rapid studies [40]. Moreover, platelets could present some bias in a comparison between healthy and diabetic subjects, as it has been reported that in T2D, these cells have an increased mean platelet volume (MPV) and are activated [41,42]. On the other hand, PBMCs are nucleated cells with a longer lifespan (6–7 months); they do not degranulate and they are easily obtainable and suitable for investigating mitochondrial respiration [43]. However it should be considered that PBMCs are an heterogeneous population of cells, and thus, their respiratory profile integrates those of lymphocytes and monocytes [44]. For this reason, it is important to monitor sample compositions and to avoid conditions of intense stress before blood sampling.

Prior studies have shown alterations in reactive oxygen species (ROS) and in mitochondrial transcriptional and protein profiles in PBMCs from diabetic patients [29,31,45], suggesting that these cells could be a useful tool to investigate T2D-related mitochondrial dysfunction. However, there is still a lack of information about functional properties of mitochondria in PBMCs, particularly in diabetic patients. It has been shown that there is a main effect of diabetes on the maximal respiration in intact cells [46], but a more detailed characterization of the role of different mitochondrial complexes in the different respiratory state is still lacking. In light of these considerations, we decided to use PBMCs and contribute to fill this gap. We hypothesized that, similarly to other tissues, PBMCs’ mitochondria from a group of postmenopausal women with T2D would display an impairment in mitochondrial function, representing an advantageous model both to investigate the causes of mitochondrial impairment and to evaluate the efficacy of therapeutic/lifestyle interventions.

We present a case-control study in which the first goal is to compare mitochondrial function parameters in PBMCs from healthy and T2D or pre-diabetic women. The second goal is to associate aspects of T2D-altered physiology (anthropometric, physical fitness, hematochemical parameters) to a characterization of blood cells’ mitochondrial function in postmenopausal women in health or with T2D.

## 2. Materials and Methods

### 2.1. Participant’s Enrollment, Inclusion Criteria and Health Data Collection

Two groups of women (9 healthy (CTRL) and 9 with a diagnosis of type 2 diabetes (N = 6) or pre-diabetic (N = 3) (T2D)) were recruited in the local area of Verona. All the participants underwent a medical examination and completed a medical history questionnaire. The following inclusion criteria were considered: age between 45 and 70, 2 or more years since menopause, diagnosis of T2D (for the diabetics group) or pre-diabetes (Hb1Ac ≥ 39 mmol/mol) [47], physical examination in the norm, normal resting ECG. Exclusion criteria were: uncompensated cardio-circulatory pathologies, respiratory diseases, renal failure, alcohol and drug abuse and neurological or orthopedic diseases limiting mobility and exercise capacity. All the participants with diagnosis of T2D were medically treated with metformin. The study conformed to the principles of the Declaration of Helsinki and was approved by the Ethics Committee for Clinical Trials of Verona and Rovigo (CESC) with protocol n° 2400CESC. All the participants gave their written informed consent.

### 2.2. Experimental Design

Before the first data collection, all the subjects underwent a familiarization session to explain all the experimental procedures that were carried out in a dry run, brief test session. All the subjects were evaluated in three different experimental sessions on three different days in the morning—day 1: blood sampling for ematochemical assays and mitochondrial function assays were carried out; day 2: DXA measurements and leg-press test; day 3: handgrip strength test and VO_2max_ determination.

### 2.3. Anthropometry and Body Composition

Body mass (BM) and stature were taken at the nearest 0.1 kg and 0.01 m, respectively, with a Tanita electronic scale BWB-800 MA (Tanita Europe B.V., Amsterdam, The Netherlands) and a stadiometer (Holtain Ltd., Crymych, Pembs, UK). Body mass index (BMI, kg/m^2^) was calculated as body mass/stature^2^. Body composition (fat mass (FM) [48], percentage FM (%FM) and lean mass (LM)) were evaluated at the whole-body (WB) and regional level (trunk, android and gynoid regions) using a total body DXA scanner (QDR Explorer W, Hologic, Bedford, MA, USA; fan-bean technology, software for Windows XP version 12.6.1) according to the manufacturer’s procedures. Accordingly, soft tissue body composition variables were also expressed either as a proportion to total body mass or normalized by square stature (Fat Mass Index (FMI); Lean Mass Index (LMI) [48]). The scanner was calibrated daily against the standard supplied by the manufacturer to avoid possible baseline drift. No special preparation was required, with the exception that participants had to wear underwear and not wear any metal accessories. The effective radiation doses involved are small (15.5 cGY cm^2^), making the technique widely applicable. Whole body scanning time was about seven minutes. Scanning and analyses were performed in the morning by the same operators (CM, VC) to ensure consistency.

### 2.4. Handgrip Strength Test

Handgrip strength (HG) of the dominant hand was assessed by a manual hand-dynamometer (Lafayette Instruments, Lafayette, IN, USA) with adjustable grip. Before starting the trials, participants performed five minutes of familiarization, standing upright with arm, forearm and wrist in a neutral position [49]. The participants squeezed the dynamometer gradually with their maximal effort for 5 s. Three maximal voluntary grips strengths were, interspersed by 1 min rest between each trial. The best trial with the dominant hand was used for statistical analysis [10,49]. HG relative to body mass (HG/BM-Kg/Kg) was also calculated to consider the involvement of body weight in the maximal muscle strength test [50].

### 2.5. Leg-Press Test

A seated leg-press device (Technogym S.p.a., Cesena, Italy) was used to measure the dynamic force production of the leg extensor muscles. Participants were positioned with a knee angle of 90 degrees, and prior to attempting one repetition maximum (1RM), they performed five minutes of familiarization where the experimenter taught the correct lifting and breathing technique at a submaximal load. Participants were instructed to grasp handles located close to the seat and to keep constant contact with the seat and backrest during leg extension to a full range of motion (180 degrees). Following a warm-up phase starting at approximately 70% of the estimated 1RM, the load was progressively increased until the participants could perform a range between 6 and 10 repetitions in each attempt that was proved suitable for this kind of population with no strength training experience [51]. Load was gradually increased by about 10–15 kg until the individual 1RM was found. The goal was to reach the desired repetitions in 3–6 attempts, interspersed by two minutes of rest between each attempt to ensure a full recovery. The Brzycki 1RM prediction equation [52] was then used to estimate the 1RM, based on the load and repetitions recorded from the participants.

### 2.6. VO_2max_

Whole-body aerobic capacity was determined by cardiopulmonary exercise test (CPET) with continuous breath-by-breath measurement of oxygen consumption during a slightly modified version of the incremental ramp test previously described [53]. The incremental test was performed on an electronically braked cycle ergometer (Excalibur Sport, Lode, Groningen, The Netherlands). The incremental test consisted of 2 min at rest and 3 min of warm-up at 30 W followed by a continuous increment, every 1 min of the workload by 10–15 W, depending on the prospective training status of each subject, until voluntary exhaustion. The latter was defined as the inability to maintain the pedaling frequency (60–80 revolutions/min), despite vigorous encouragement by the experimenters. Workload continuous increment was set a priori so that the subjects achieved voluntary exhaustion in 10–12 min. The ergometer was associated with a metabolic cart (Quark b^2^, Cosmed, Rome, Italy) that allowed for continuous, breath-by-breath measures of gas exchange (at the mouth), ventilation and heart rate (HR). Before each test, the gas analyzers and the turbine flow meter of the system were calibrated, following the manufacturer’s instructions, by using a reference gas mixture of known concentration (FO_2_: 0.16; FCO_2_: 0.05; N_2_ as balance) and a 3.0-litre calibrated syringe.

VO_2max_ was assessed as the mean of the O_2_ values occurring in the last 30 s of the constant work-rate test before the interruption. Furthermore, VO_2max_ was normalized by body mass (VO_2max/kg_).

### 2.7. Blood Sampling

Fasting venous blood samples were drawn from an antecubital vein in the morning (7:00–9:00 a.m.) after a 12 h overnight fast in accordance with the recommendations of the European Federation of Clinical Chemistry and Laboratory Medicine (EFLM) [54]. Participants were instructed to abstain from strenuous physical activity for 48 h before the blood samples were taken.

### 2.8. Hematological Testing

All the samples were processed for routine hematological testing immediately after collection (<15 min) on the Advia 2120i hematology system (Siemens Healthcare Diagnostics, Deerfield, IL, USA) using standard local procedures at GB Rossi Hospital, Verona, Italy. The parameters tested included red blood cells count (RBC), hematocrit (HCT), hemoglobin (HGB), mean red cell volume (MCV), mean red cell hemoglobin content (MCHC), red blood cell distribution width (RDW), white blood cells (WBC) count, and WBC differential, including lymphocytes, monocytes, neutrophils, eosinophils, basophils and large unstained cells, platelet count, and mean platelet volume. The instrument was calibrated against appropriate proprietary reference standard material and verified with the use of proprietary controls.

### 2.9. Clinical Chemistry and Immunochemistry Test

The clinical chemistry and immunochemistry tests were performed on serum aliquots on the instrument Cobas^®^ 6000 < c501 > and < e601 > module (Roche Diagnostics GmbH, Penzberg, Germany), according to the manufacturer’s specifications and using proprietary reagents. The panel of tests included the following markers: Hb1Ac, glucose, C-reactive protein (CRP), total cholesterol (Chol_TOT_), HDL cholesterol (Chol_HDL_), LDL cholesterol (Chol_LDL_), and triglycerides (TG). The instrument was calibrated against appropriate proprietary reference standard materials and verified with the use of proprietary quality controls. Plasmatic levels of total glutathione (GSHt) were determined using a colorimetric assay (Glutathione Assay Kit–Cayman Chemical, Ann Arbor, MI, USA) according to the manufacturer’s instructions.

### 2.10. PBMCs Purification

PBMCs were isolated from whole blood collected in 2X9 mL K2-EDTA Vacuettes. All the procedures were performed at room temperature. The samples were kept under gentle agitation for 30 min before proceeding with the subsequent centrifugation steps. Whole blood was diluted 1:1 with RPMI-1640 medium (Sigma-Aldrich, Milano, Italy) and loaded on a 50 mL LeucoSep tube (Greiner, GmbH) previously prepared with 15 mL of Ficoll (Cytiva, Uppsala, Sweden AB). The PBMCs cell layer was obtained by centrifugation at 800 g for 10 min in Heraeus 2704 centrifuge with swinging-bucket rotor and transferred to a new 50 mL Falcon (Becton Dickinson, Franklin Lakes, NJ, USA) tube. Contaminant platelets were removed with two subsequent washing steps in RPMI (30 mL), each at 100 g for 10 min. The final pellet was resuspended in MiR05 medium, and cell concentration and composition were determined using a Sysmex-XN1000 analyzer (Supplementary Figure S1).

### 2.11. High Resolution Respirometry

**Figure 1:**
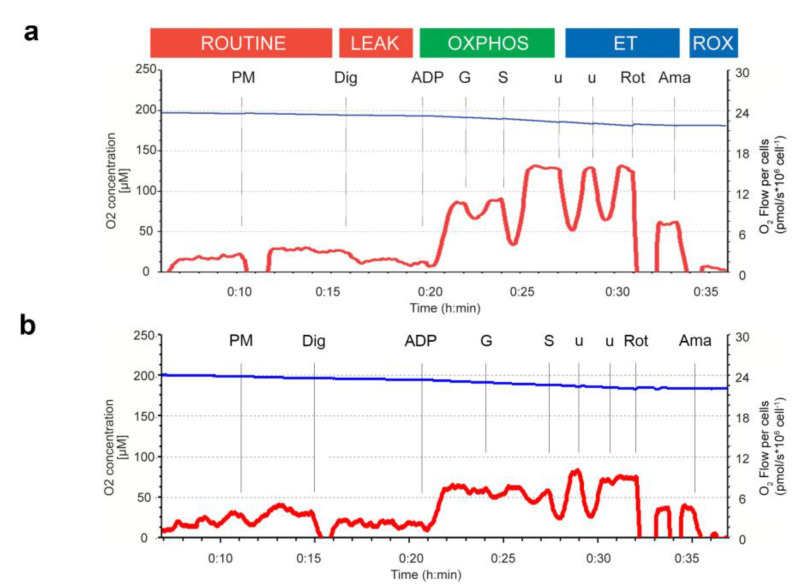
(**a**,**b**) Representative respirometry traces following high-resolution respirometry protocol applied to permeabilized PBMCs obtained from CTRL (**a**) and T2D (**b**) samples. Blue line: O_2_ concentration over time; Red line: cell-specific O_2_ flow (JO_2_), calculated as the negative time derivative of O_2_ concentration, corrected for instrumental background, and expressed as pmol/s*10^6^ cells^−1^. For titration steps and SUIT protocol, see the Methods section. Abbreviations: ROUTINE, state; LEAK, state; OXPHOS, state; ET, Electron transfer pathway; ROX, residual oxygen consumption; PM, pyruvate and malate; PMG, pyruvate, malate and glutamate (CI-linked electron transfer); PMGS, pyruvate, malate, glutamate, and succinate (CI&CII-linked electron transfer); Dig, digitonin; ADP, adenosine diphosphate; u, uncoupler, CCCP titration; Rot, rotenone; Ama, Antimycin A.

High resolution respirometry (HRR) analysis was performed on permeabilized PBMCs as previously described [55,56]. Purified PBMCs were loaded in the O_2_k chambers (3 * 10^6^ cells/chamber) of an Oxygraph-2k (Oroboros Instruments GmbH, Innsbruck, Austria) for HRR, and a Substrate Uncoupler Inhibitor Titration (SUIT) protocol for permeabilized cells was used for analysis [56]. Mitochondrial respiration was analyzed in different respiratory states: the ROUTINE based on endogenous substrates and ADP levels, the dissipative fraction of oxygen consumption was recorded in the LEAK corresponding to the oxygen used to restore dissipative proton-leak, the OXPHOS state corresponding to active phosphorylative oxidation stimulated by saturating ADP and substrates to sustain complex I (pyruvate (P) and malate (M) (PM), and glutamate (G) (PMG)) and complex II (succinate (S) (PMGS)) (Figure 1a,b). 

For the ROUTINE, pyruvate 5 mM and malate 0.5 mM were added. Then, cells were permeabilized with Digitonin (Dig) (10 mg/mL stock in DMSO) to allow specific substrates to enter the cells and to induce the transition to the LEAK state. The OXPHOS_PM_ state was triggered by addition of adenine diphosphate (ADP) 1 mM to elicit oxidative phosphorylation. Glutamate (Sigma Aldrich, Milano, Italy) 10 mM was added to complete cI-linked respiration (OXPHOS_PMG_). Succinate 10 mM was then added to induce cII-linked respiration (OXPHOS_PMGS_). Next, carbonyl cyanide m-chlorophenyl hydrazine (CCCP) (Sigma Aldrich, Milano, Italy) or uncoupler titrations (0.25 μM per step) were used to reach maximum uncoupled respiration, determining the maximal electron transfer capacity (ET state). Finally, rotenone (Rot) 0.5 μM was added to inhibit cI; thus, ET sustained by cII activity was evaluated. Lastly, Antimycin A 2.5 μM was used to inhibit cIII, and residual oxygen consumption (ROX) was measured. Data were recorded and analyzed with DatLab6 software (Oroboros Instruments GmbH, Innsbruck, Austria) and expressed as oxygen flow normalized per cell number (JO2–pmol O2/sec*10^6^ cells^−1^) or flux control ratio (relative to ET) (FCR). Net OXPHOS and ET capacity factors were calculated by correcting the OXPHOS and ET values for the LEAK respiration values (OXPHOS_PMGS_-LEAK_PM_; ET- LEAK_PM_). The ET-reserve capacity was obtained by subtracting the ROUTINE from ET values.

### 2.12. Data Analysis and Statistics

All the data were collected and organized in Excel (Microsoft). Statistical analysis was performed with Prism 8 (GraphPad Software, San Diego, CA, USA) and SPSS Statistics 27.0 (IBM Corp, Armonk, NY, USA). Normality of data distribution was verified using the Shapiro–Wilk test. When criteria for normality were not met, non-parametric Mann–Whitney test was performed (this occurred only for the CRP marker). The comparison of mean values between groups were conducted with unpaired Student’s *t* test or two-way ANOVA with Tukey post hoc correction. Pearson correlation was used to examine the relationship between variables. Significance was set at an α level of 0.05, and results are presented as mean ± st.dev. The sample size was determined using G^∗^Power software (ver 3.1.9.6) [57] to ensure, for key parameters of mitochondrial respirometry assays, sufficient power (1–β = 0.80) to detect significant differences between groups using two-way ANOVA. A priori determination of the sample size was found to be 8 per group.

## 3. Results

### 3.1. Anthropometric Characteristics and Body Composition

A case-control comparison of demographic and anthropometric characteristics of participating women from CTRL and T2D groups is shown in Table 1. The T2D group had higher body mass (BM) and BMI (+16.2% and +17.2%, respectively) compared to the CTRL group (Table 1). The android/gynoid fat mass ratio was significantly higher in the T2D group (+20%; *p* = 0.005), and a tendency was observed for trunk fat expressed in percentage (+17%; *p =* 0.05).

### 3.2. Blood Biomarkers

Hematological and hematochemical analysis from fasting blood samples are reported in Table S1 and Table 2. The blood cell profile was similar between the two groups, showing no relevant differences (Table S1). Participants of the T2D group had higher levels of Hb1Ac (+31.3%; *p =* 0.002), glucose (+40%; *p =* 0.008) with a tendency toward higher levels of the inflammatory marker C-reactive protein (CRP +120%; *p =* ns) (Table 2). The diabetic group also showed alterations of the lipidic profile with a characteristic dyslipidemia signature determined by lower HDL cholesterol (Chol_HDL_ −35%; *p =* 0.005) and higher triglycerides (TG +63%; *p =* 0.03) compared to CTRL subjects (Table 2). Consistently, the TG/HDL ratio is higher in the T2D group (+125%; *p =* 0.03) (Table 2).

### 3.3. Cardio-Respiratory Function and Fitness

Data obtained from the analysis of cardiopulmonary exercise tests (CPET) comparing T2D and CTRL women are reported in Table 3. Absolute VO_2max_ and VO_2max_ adjusted for body mass (VO_2max/kg_) values were similar between the two groups or tendentially lower in the T2D group (VO_2max/kg_ −15%; *p =* ns). The participants in the T2D group had a lower HR_max_ (10%, *p =* 0.01) compared to CTRL group (Table 3).

### 3.4. Muscle Strength

Functional strength of lower- and upper-limb muscles was tested using a leg-press and a hand-dynamometer, respectively. The values of absolute leg-press 1RM and 1RM normalized for body mass (1RM/BM) showed no differences between the two groups (Table 3). The HG strength was similar between the two groups, but HG values normalized per body mass (HG/BM) were significantly reduced in the T2D group (−23%; *p =* 0.02).

### 3.5. Mitochondrial Function

**Figure 2:**
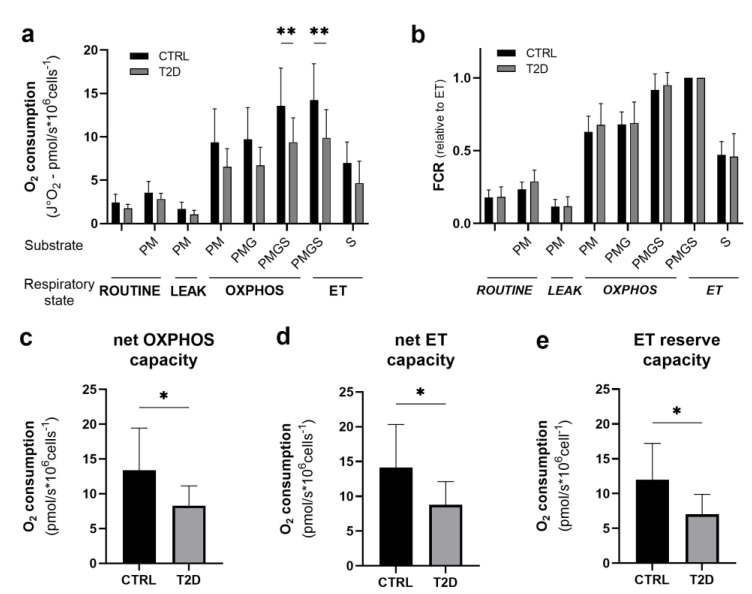
Analysis of mitochondrial function in PBMCs from CTRL and T2D samples. (a) Quantitative analysis of the rate of oxygen consumption (expressed as O_2_ pmol/s per million cells) in the ROUTINE, LEAK, OXPHOS and ET respiratory states in PBMCs from CTRL and T2D blood samples. (b) Qualitative analysis of data of mitochondrial function in permeabilized PBMCs expressed relative to ET (flux control ratio, FCR) in the respiratory states analyzed (*ROUTINE, LEAK, OXPHOS* and *ET*). (c) Net OXPHOS capacity, index of ADP stimulation, for CTRL and T2D PBMC cells calculated as (OXPHOS–LEAK). (d) Net ET capacity for CTRL and T2D PBMC cells calculated as (ET–LEAK). (e) ET reserve capacity, index of the potential of substrate uptake and ADP stimulation, for CTRL and T2D PBMC cells calculated as (ET–ROUTINE). Data are reported as mean ± st.dev (CTRL N = 9; T2D N = 9); * *p* < 0.05, ** *p* < 0.01.

Mitochondrial function was analyzed in permeabilized PBMCs isolated from freshly collected blood samples (Figure 1 and Figure 2). Mitochondrial respiration was analyzed in different respiratory states: the ROUTINE based on endogenous substrates and ADP levels, the dissipative fraction of oxygen consumption was recorded in the LEAK corresponding to the oxygen used to restore dissipative proton-leak, and the OXPHOS state corresponding to active phosphorylative oxidation stimulated by saturating ADP and substrates to sustain complex I (pyruvate, malate, and glutamate) and complex II (succinate) (Figure 1a,b).

To analyze differences between the mean values of the various respiratory states of the two groups of subjects, two-way ANOVA of specific oxygen flow values (J°O_2_-pmol O_2_/s*10^6^ cells^−1^) was performed (Figure 1c). Overall, we found a significant difference between groups (ANOVA *p* < 0.0001); furthermore, samples from the T2D group showed reduced maximal phosphorylative capacity (OXPHOS_PMGS_ −30%; *p =* 0.006) and maximal non-coupled respiratory capacity (ET_PMGS_ −30%; *p =* 0.004) (Figure 2a, Table 4). We also evaluated the relative contribution of mitochondrial complexes and substrate combinations using a qualitative analysis of data expressed as flux control ratios (FCR), in which data are expressed relatively to the maximal non-coupled oxidative capacity (ET). In this case, the means of two groups show no relevant difference (Figure 2b, Table 4). Net and reserve capacities resulted to be significantly reduced in mitochondria from the T2D compared to the CTRL group (net OXPHOS capacity −40%, *p =* 0.03; net ET capacity −38%, *p =* 0.03; and ET reserve capacity −41%, *p =* 0.02) (Figure 2c–e, Table 4).

### 3.6. Glutathione Antioxidant Pool

One of the causes related to impaired mitochondrial function in T2D has been suggested to rely on the accumulation of oxidative stress. The antioxidant glutathione (GSHt) is a primary element in the ROS scavenging system. Hence, we tested the abundance of the antioxidant GSHt in plasma samples from the two groups. In T2D samples, there is an important reduction of the GSHt pool (−38%; *p =* 0.04) (Table 2).

### 3.7. Correlation Analysis

To investigate the associations between individual values of parameters for the whole study group, Pearson correlation was performed, and significant associations are reported in Table 4. The fraction of Hb1Ac is associated with age, CRP, TG levels and with fat deposits of the android region (TRUNK PFAT and ANDROID/GYNOID). Furthermore, Hb1Ac is negatively associated with Chol_HDL_, HR_max_, and with mitochondrial activity (LEAK, OXPHOS_PMGS_, ET, net ET, and ET reserve capacity) (Table 4). Mitochondrial respiration is associated with several physiological parameters in different respiratory states (Table 4): it is negatively associated with TG levels in the non-stimulated ROUTINE and LEAK_PM_ respiratory states, and as mentioned above, it is negatively associated with Hb1Ac. Mitochondrial respiration is also negatively associated with fat deposits (WB PFAT and TRUNK PFAT) in OXPHOS, ET and LEAK, OXPHOS, ET, net capacity, and ET reserve capacity (Table 4). Moreover, mitochondrial oxidative capacity (OXPHOS, net OXPOS capacity, netET capacity and ET reserve capacity) is positively associated with whole body maximal oxygen consumption (VO_2max_) (Table 4).

An unbiased grouping of all the subjects according to hierarchical clustering confirms the distribution of the subjects in the two groups (Supplementary Figure S2).

## 4. Discussion

Here, we compared mitochondrial function in samples from two groups of overweight postmenopausal women (i.e., CTRL vs. T2D), together with a wide panel of parameters describing body composition, blood biomarkers, cardiorespiratory physiology and strength performance. Our purpose was to verify whether PBMCs from the T2D group would display an impairment in mitochondrial function, and thus could represent an advantageous model both to investigate the causes of mitochondrial impairment and to evaluate the efficacy of therapeutic/lifestyle interventions at a systemic level. Several significant differences were found between the two groups: as expected in levels of glycated hemoglobin and fasting glucose, but also in body composition and fat deposit distribution, lipidic profile and levels of mitochondrial respiration. Alterations in most of these parameters are well established hallmarks of T2D condition: carbohydrate and lipid metabolism are closely intertwined, and elevated circulating and intracellular lipids may disrupt glucose homeostasis.

A wide range of defects in mitochondrial function have been associated with T2D: defects in mitochondrial bioenergetics, biogenesis and dynamic processes, and in particular, defects in mitochondrial bioenergetics affect fatty acids transport and metabolism, cellular metabolism, and ROS accumulation [34,58]. We report an overall reduction in mitochondrial respiration in PBMCs that becomes significant during the active ADP-stimulated oxidative phosphorylation sustained by complex I (OXPHOS_PMG_) and its combination with complex II (OXPHOS_PMGS_) and the non-coupled maximal respiratory capacity (ET). Consistently, indexes such as net OXPHOS capacity, net ET capacity and ET reserve capacity are significantly reduced in T2D samples, suggesting that PBMCs cells from T2D subjects are less capable of conducting ADP phosphorylation. However, analysis of flux control ratios (FCRs), describing the relative contribution of single mitochondrial complexes or their combinations to cellular respiration, failed to point out any significant qualitative difference between mitochondria of CTRL and T2D groups. Reduced expression of a master gene of mitochondrial biogenesis (*PGC1A*) has been reported in skeletal muscle samples from T2D patients, and it has been associated with reduced expression of metabolic and mitochondrial gene sets [29,59]. In addition, genes involved in the regulation of mitochondria dynamics display altered patterns of expression, such as the mitochondrial fusion gene *MFN2* that is expressed at lower levels in insulin-resistant individuals [60]. In PBMCs, alterations at different levels have been associated with T2D: ROS accumulation, mitochondrial transcriptional and protein profile [1]. Several studies focused on defects of mitochondrial function in T2D, but despite abundant data, it is still unclear whether perturbations of mitochondrial functions are a cause or a consequence of insulin resistance [61,62]. Altered activity of mitochondria was reported in the skeletal muscle of T2D patients, showing important reduction (−40%) of complex I activity and lower oxidative phosphorylation activity of the ET system [30,63,64,65,66,67,68], suggesting that T2D-related defects in mitochondrial bioenergetics may result, at least in part, from a reduction in mitochondrial number. Although skeletal muscle plays a pivotal role in insulin-stimulated glucose uptake (up to 80%), mitochondrial dysfunction has consistently been associated with T2D in multiple tissues (liver, white and brown adipose tissue, brain, cardiac muscle and stem cells) [25,34]. It was already shown that there is an association between diabetes and maximal mitochondrial respiration in intact PBMCs [69]; however, in this study, we pushed the investigation of mitochondrial defects a little further, trying to characterize the contribution of mitochondrial complexes to cellular bioenergetics (mainly complexes I and II). Our data suggest that in diabetes, mitochondrial oxidative capacity is also impaired in blood cells, further supporting the idea that T2D-linked alterations of mitochondrial function may be systemic. More studies are required to investigate the role of other complexes and pathways (i.e., fatty acids oxidation) and to verify whether this impairment rises from a reduction of the expression of single complexes (or their subunits), of mitochondrial content or of dynamic processes.

Regarding the associations between parameters of mitochondrial function and markers of dysglycemia, dyslipidemia and physical fitness, we found consistent association between mitochondrial function and the levels of Hb1Ac, circulating lipids (TG and Chol_HDL_) and between fat distribution (abdominal fat) and cardiorespiratory fitness (VO_2max_/kg). These associations confirm the intertwined links between impairment in the control of glycemia, dyslipidemia, reduced physical performance and mitochondrial function, representing the pillar of cellular metabolism. We are still far from identifying the single element that leads to the onset of this multifactorial disease, but we suggest that more attention should be focused on the continual increase in Hb1Ac. Likewise the use of markers of mitochondrial function should be further validated and may be implemented to monitor and early diagnose a decay in an individual’s cellular metabolism. Such markers could help to identify metabolic changes before the disease becomes irreversible and at a stage where therapeutic and lifestyle intervention could be more effective, thus reducing the burden of the health and economic costs of this epidemic.

Between the CTRL and T2D groups, dysregulation of lipid metabolism is evident, with higher levels of circulating TGs and lower Chol_HDL_, characteristic features of diabetes associated with an increased risk of cardiovascular disease [3]. Lipid metabolism is regulated at several levels: the adipose tissue is the main storage system for lipids; it releases lipids (mainly TGs) into circulation following stimulation by the liver. The hormonal and metabolic changes associated with menopause are followed by fat redistribution toward the upper body, including the abdominal/visceral area [70]; however, this tendency to accumulate fat in the abdominal area is exacerbated in T2D [71,72]. To characterize this shift in fat distribution, we considered an index of ectopic fat deposition, the ratio between the android vs. gynoid region fat mass, and the percent of fat in the trunk region. According to the literature [72], the T2D group had higher ectopic fat deposits in the abdominal areas as compared to the controls. Associations were found between these parameters of fat deposition with hematological markers of dyslipidemia and glycated hemoglobin, reinforcing the idea that hypertrophy of abdominal fat is predictive of T2D [71,72]. Values relating to abdominal fat storage (i.e., TRUNK PFAT) are also negatively associated with mitochondrial respiration parameters. These data are consistent with previous studies that reported a negative correlation between PBMCs ET reserve capacity (“spare respiratory capacity” in the original) and BMI in overweight and obese older adults [73]. Furthermore, mitochondrial oxidative capacity (skeletal muscle biopsies) was found to be negatively associated with markers of systemic inflammation, increasing BMI, adipose tissue and abdominal obesity [69,74]. Taken together, these observations highlight how adipose tissue accumulation is closely related to systemic mitochondrial dysfunction.

T2D has also been associated with progressive loss of muscle mass and function, and this reduction affects its metabolic capacity [75,76,77]. Parameters describing muscular mass and its functional properties in subjects with diabetes were clearly lower than those of healthy subjects [77]. However, in our study, we found no significant differences between the two groups regarding parameters relating to skeletal muscle mass or muscle strength, except for handgrip strength expressed relative to body mass. This apparent discrepancy may be due to the fact that our subjects were all women, and it has been recently reported that no decline in muscle mass and strength is observed in middle aged/older women with T2D [78].

In the pathogenesis of T2D, the accumulation of oxidative stress has been implicated, and it can be due either to increased production of oxidant species or to a reduction in enzymatic and non-enzymatic antioxidants in diabetic patients. Mitochondria are the main source and targets of oxidative reactions, and the antioxidant glutathione is a key element in the ROS scavenging system. Here, we report that in postmenopausal women with T2D, the total pool of GSHt in serum is reduced as compared to women from the CTRL group, suggesting that the ROS scavenging system may be less efficient in T2D patients, and this could lead to increased accumulation of reactive species. Glutathione is a primary antioxidant, and its total pool has been reported in several studies to be reduced in T2D patients [79]. GSHt is ubiquitous in the cells and is transported in the mitochondria, where it plays a role as a redox regulator of ETS complexes involved in oxidative phosphorylation [80]. This may be a relevant factor to explain the reduced levels of mitochondrial activity in our T2D samples: several findings in different cell types showed a dependence of mitochondrial function on GSHt levels [81,82].

We recognize that the choice of PBMCs is targeted to a systemic picture of mitochondrial function/dysfunction and that tissue-specific alterations may occur in T2D. However, the consistency of our findings with the literature relating to other tissues (PBMCs, skeletal muscle, liver) suggests that data are rather representative of widespread mitochondrial dysfunction in various organs. We used only the respirometry to assess mitochondrial impairment; however, the parallel analysis of other biomarkers of mitochondrial content, or of oxidative stress, may have been helpful to strengthen the interpretation in results and to disentangle the deficiency in a specific complex or the reduced number of mitochondria. Although PBMCs are composed of different cell types (monocytes and lymphocytes), we found no significant difference in PBMCs composition between CTRL and T2D samples (Supplementary Figure S1). Finally, the small number of participants in our study may reduce the generalizability of our results. Despite these limitations, the data show a good consistency both with the literature and within the study presented.

## 5. Conclusions

Firstly, our data indicate that in overweight postmenopausal women, T2D-related mitochondrial activity defects can also be detected in PBMCs cells. This makes this cell population a suitable model to investigate the mechanisms underlying mitochondrial dysfunction in T2D and to identify possible targets for therapeutical or interventional approaches. The consistency of the observations collected with previous studies also performed on other tissues suggests that PBMCs may be a good and promising alternative to tissue biopsies for future investigations of mitochondrial function in T2D.

Secondly, we found an association between mitochondrial respiration parameters and aspects of whole body physiology at different levels (anthropometrics, cardio-respiratory fitness, hematochemical markers), suggesting that there is a link between mitochondrial and systemic fitness. Then, in addition to confirming the presence of several hallmarks of T2D, this study points to the use of PBMCs as an alternative source of mitochondria to investigate mitochondrial function in T2D, as well as in other pathologies.

## Figures and Tables

**Table 1:** Anthropometric characteristics of body composition and fat distribution of the participants. Data relative to each parameter were compared using unpaired Student’s *t* test. Data are expressed as mean ± st.dev; (CTRL *N* = 9; T2D *N* = 9).

Description	CTRL (N = 9)	T2D (N = 9)	∆(T2D vs. CTRL) (%)	*p* Value (Unpaired Student’s *t* Test)
Age	54.1 ± 3.8	60.9 ± 4.8		0.004
Ethnicity	Caucasian	Caucasian		
Height (cm)	162 ± 0.05	162 ± 0.07	-	ns
BM (kg)	67.8 ± 16.1	78.5 ± 16.8	+16.2	ns
BMI (Kg/m^2^)	25.6 ± 5.2	30.0 ± 5.9	+17.2	ns
LEAN MASS (kg)	39.7 ± 6.7	44.6 ± 7.9	+13.7	ns
LMI (Kg/m^2^)	15.1 ± 1.9	17.1 ± 2.6	+13	ns
FAT MASS (Kg)	24.9 ± 9.4	30.8 ± 9.7	+23	ns
FMI (Kg/m^2^)	9.4 ± 3.2	11.8 ± 3.7	+25	ns
WB FAT(%)	36.4 ± 5.7	38.3 ± 4.4	+5	ns
ANDROID/GYNOID	0.45 ± 0.04	0.54 ± 0.07	+20	0.005
TRUNK FAT(%)	33.8 ± 7.2	39.8 ± 4.5	+17	0.05

**Table 2:** Values of circulating blood markers (glycated hemoglobin—Hb1Ac, C-reactive protein—CRP, total cholesterol—Chol_TOT_, HDL cholesterol—Chol_HDL_, LDL cholesterol—Chol_LDL_, triglycerides—TG, total glutathione—GSHt). Data for each parameter were compared between the two groups using an unpaired Student’s *t* test. Data are expressed as mean ± st.dev (CTRL N = 9; T2D N = 9).

Description	CTRL (N = 9)	T2D (N = 9)	∆(T2D vs. CTRL) (%)	*p* Value (Unpaired Student’s *t* Test)
Hb1Ac (mmol/mol)	34.4 ± 1.7	45.2 ± 7.6	+31.3	0.002
Glucose (fasting)	4.4 ± 0.7	6.2 ± 1.5	+40	0.008
CRP (mg/L)	1 ± 0	2.2 ± 2.3	+120	ns #
Chol_TOT_ (mmol/L)	5.1 ± 0.9	4.9 ± 1.2	−4	ns
Chol_HDL_ (mmol/L)	1.9 ± 0.4	1.4 ± 0.3	−35	0.005
Chol_LDL_ (mmol/L)	2.9 ± 0.4	3.3 ± 0.7	+13	ns
TG (mmol/L)	0.92 ± 0.4	1.5 ± 0.7	+63	0.03
TG/Chol_HDL_ ratio	0.54 ± 0.33	1.21 ± 0.75	+125	0.03
GSHt (%)	100 ± 35.7	61.8 ± 31.8	−38	0.04

# Unpaired Mann–Whitney test.

**Table 3:** Values of cardiorespiratory fitness (CPET test) and strength parameters (1RM and HG). The following parameters were considered: maximal cardio-respiratory capacity (VO_2max_)_,_ peak power output (PPO_max_), maximal heart rate (HR_max_), one-repetition maximum for the leg extensor muscles (1RM), and grip strength (HG). Data for each parameter were compared between the two groups using an unpaired Student’s *t *test. Data are expressed as mean ± st.dev (CTRL *N* = 9; T2D *N* = 9).

Description	CTRL (N = 9)	T2D (N = 9)	∆(T2D vs. CTRL) (%)	*p* Value (Unpaired Student’s *t* Test)
VO_2max_ (mL)	1857.4 ± 202.5	1856.3 ± 256.0		ns
VO_2max_/Kg (mL/Kg)	28.8 ± 7.1	24.5 ± 5.6	−15	ns
PPO_max_ (Watt)	167.7 ± 18.5	149.1 ± 25.6	−12	ns
HR_max_ (bpm)	167.2 ± 6.7	151.3 ± 15.0	−10	0.01
1RM (Kg)	168.1 ± 41.1	176.7 ± 48.7	+5	ns
1RM/BM	2.53 ± 0.61	2.27 ± 0.46	−11	ns
HG (Kg)	30.9 ± 4.7	29.6 ± 5.8	−4	ns
HG/BM (Kg/Kg)	0.47 ± 0.09	0.38 ± 0.04	−23	0.02

**Table 4:** Correlation analysis. Pearson’s correlation analysis between individual values of the factors considered in the whole study.

Factor		r	*p* Value
*Blood markers*			
Hb1Ac	Age	0.557	0.016
	CRP	0.618	0.006
	Chol_HDL_	−0.553	0.017
	TG	0.815	<0.0001
	GSTt	0.544	0.020
	HR_max_	−0.651	0.003
	Android/Gynoid	0.570	0.013
	Trunk PFAT	0.503	0.034
	LEAK_PM_	−0.476	0.046
	OXPHOS_PMGS_	−0.502	0.034
	ET	−0.529	0.024
	ET reserve capacity	−0.501	0.034
*Mitochondrial activity*			
ROUTINE	TG	−0.490	0.039
ROUTINE_PM_	HDL	0.487	0.041
LEAK_PM_	Hb1Ac	−0.476	0.046
	Chol_HDL_	0.565	0.015
	TG	−0.533	0.023
	TRUNK_PFAT	−0.556	0.017
OXPHOS_PM_	VO_2max_/kg	0.477	0.045
	HG/BM	0.559	0.016
	WB PFAT	−0.527	0.025
	TRUNK PFAT	−0.606	0.008
OXPHOS_PMG_	HG/BM	0.552	0.018
	WB PFAT	−0.529	0.024
	TRUNK PFAT	−0.550	0.018
OXPHOS_PMGS_	Hb1Ac	−0.502	0.034
	Chol_HDL_	0.518	0.028
	VO_2max_/kg	0.464	0.052
	HG/BM	0.608	0.007
	WB PFAT	−0.508	0.032
	TRUNK PFAT	−0.602	0.008
ET_PMGS_	Hb1Ac	−0.529	0.024
	Chol_HDL_	0.491	0.039
	HG/BM	0.579	0.012
	WB PFAT	−0.481	0.043
	TRUNK PFAT	−0.570	0.014
ET_S_	Hb1Ac	−0.526	0.025
netOXPHOS capacity	Chol_HDL_	0.564	0.015
	VO_2max_/kg	0.504	0.033
	HG/BM	0.615	0.007
	TRUNK PFAT	−0.627	0.005
netET capacity	Hb1Ac	−0.467	0.051
	Chol_HDL_	0.574	0.013
	VO_2max_/kg	0.470	0.049
	HG/BM	0.591	0.010
	TRUNK PFAT	−0.615	0.007
ET reserve capacity	Hb1Ac	−0.501	0.034
	Chol_HDL_	0.597	0.009
	TG	−0.460	0.055
	VO_2max_/kg	0.488	0.040
	HG/BM	0.613	0.007
	TRUNK PFAT	−0.649	0.004

## Data Availability

Data are contained within the article or Supplementary Material.

## Supplementary Materials

The following supporting information can be downloaded at: https://www.mdpi.com/article/10.3390/biomedicines11010121/s1, Figure S1: Graphical representation of the types of cells present in purified PBMCs samples.; Figure S2: Tree diagram of hierarchical grouping considering markers for all the participants; Table S1: Hematological and hematochemical parameters obtained from blood samples of the participants.

## References

[1] Unnikrishnan R., Pradeepa R., Joshi S.R., Mohan V. (2017). Type 2 Diabetes: Demystifying the Global Epidemic. Diabetes.

[2] Krako Jakovljevic N., Pavlovic K., Jotic A., Lalic K., Stoiljkovic M., Lukic L., Milicic T., Macesic M., Stanarcic Gajovic J., Lalic N.M. (2021). Targeting Mitochondria in Diabetes. Int. J. Mol. Sci..

[3] American Diabetes A. (2014). Diagnosis and classification of diabetes mellitus. Diabetes Care.

[4] Greiner G.G., Emmert-Fees K.M.F., Becker J., Rathmann W., Thorand B., Peters A., Quante A.S., Schwettmann L., Laxy M. (2020). Toward targeted prevention: Risk factors for prediabetes defined by impaired fasting glucose, impaired glucose tolerance and increased HbA1c in the population-based KORA study from Germany. Acta Diabetol..

[5] Zhang X., Gregg E.W., Williamson D.F., Barker L.E., Thomas W., Bullard K.M., Imperatore G., Williams D.E., Albright A.L. (2010). A1C level and future risk of diabetes: A systematic review. Diabetes Care.

[6] Wells J.C.K. (2019). The diabesity epidemic in the light of evolution: Insights from the capacity-load model. Diabetologia.

[7] Wu L., Parhofer K.G. (2014). Diabetic dyslipidemia. Metabolism.

[8] Verges B. (2015). Pathophysiology of diabetic dyslipidaemia: Where are we?. Diabetologia.

[9] Wang Y.L., Koh W.P., Talaei M., Yuan J.M., Pan A. (2017). Association between the ratio of triglyceride to high-density lipoprotein cholesterol and incident type 2 diabetes in Singapore Chinese men and women. J. Diabetes.

[10] Hu S., Gu Y., Lu Z., Zhang Q., Liu L., Meng G., Yao Z., Wu H., Bao X., Chi V.T.Q. (2019). Relationship Between Grip Strength and Prediabetes in a Large-Scale Adult Population. Am. J. Prev. Med..

[11] Qin H., Chen Z., Zhang Y., Wang L., Ouyang P., Cheng L., Zhang Y. (2020). Triglyceride to high-density lipoprotein cholesterol ratio is associated with incident diabetes in men: A retrospective study of Chinese individuals. J. Diabetes Investig..

[12] Reusch J.E., Bridenstine M., Regensteiner J.G. (2013). Type 2 diabetes mellitus and exercise impairment. Rev Endocr Metab Disord.

[13] Regensteiner J.G., Bauer T.A., Reusch J.E., Quaife R.A., Chen M.Y., Smith S.C., Miller T.M., Groves B.M., Wolfel E.E. (2009). Cardiac dysfunction during exercise in uncomplicated type 2 diabetes. Med. Sci. Sports Exerc..

[14] Gulsin G.S., Henson J., Brady E.M., Sargeant J.A., Wilmot E.G., Athithan L., Htike Z.Z., Marsh A.M., Biglands J.D., Kellman P. (2020). Cardiovascular Determinants of Aerobic Exercise Capacity in Adults With Type 2 Diabetes. Diabetes Care.

[15] Laukkanen J.A., Kurl S., Salonen R., Rauramaa R., Salonen J.T. (2004). The predictive value of cardiorespiratory fitness for cardiovascular events in men with various risk profiles: A prospective population-based cohort study. Eur. Heart J..

[16] Joseph J.J., Echouffo-Tcheugui J.B., Golden S.H., Chen H., Jenny N.S., Carnethon M.R., Jacobs D., Burke G.L., Vaidya D., Ouyang P. (2016). Physical activity, sedentary behaviors and the incidence of type 2 diabetes mellitus: The Multi-Ethnic Study of Atherosclerosis (MESA). BMJ Open Diabetes Res. Care.

[17] Wahid A., Manek N., Nichols M., Kelly P., Foster C., Webster P., Kaur A., Friedemann Smith C., Wilkins E., Rayner M. (2016). Quantifying the Association Between Physical Activity and Cardiovascular Disease and Diabetes: A Systematic Review and Meta-Analysis. J. Am. Heart Assoc..

[18] Abdullah A., Stoelwinder J., Shortreed S., Wolfe R., Stevenson C., Walls H., de Courten M., Peeters A. (2011). The duration of obesity and the risk of type 2 diabetes. Public Health Nutr..

[19] Logue J., Walker J.J., Colhoun H.M., Leese G.P., Lindsay R.S., McKnight J.A., Morris A.D., Pearson D.W., Petrie J.R., Philip S. (2011). Do men develop type 2 diabetes at lower body mass indices than women?. Diabetologia.

[20] Kannel W.B. (2002). The Framingham Study: Historical insight on the impact of cardiovascular risk factors in men versus women. J. Gend Specif. Med..

[21] Kenchaiah S., Vasan R.S. (2015). Heart Failure in Women--Insights from the Framingham Heart Study. Cardiovasc Drugs Ther..

[22] Wexler D.J., Grant R.W., Meigs J.B., Nathan D.M., Cagliero E. (2005). Sex disparities in treatment of cardiac risk factors in patients with type 2 diabetes. Diabetes Care.

[23] Rossi M.C., Cristofaro M.R., Gentile S., Lucisano G., Manicardi V., Mulas M.F., Napoli A., Nicolucci A., Pellegrini F., Suraci C. (2013). Sex disparities in the quality of diabetes care: Biological and cultural factors may play a different role for different outcomes: A cross-sectional observational study from the AMD Annals initiative. Diabetes Care.

[24] Wedisinghe L., Perera M. (2009). Diabetes and the menopause. Maturitas.

[25] Gonzalez-Franquesa A., Patti M.E. (2017). Insulin Resistance and Mitochondrial Dysfunction. Adv. Exp. Med. Biol..

[26] Ruegsegger G.N., Creo A.L., Cortes T.M., Dasari S., Nair K.S. (2018). Altered mitochondrial function in insulin-deficient and insulin-resistant states. J. Clin. Invest..

[27] Pacheu-Grau D., Rucktaschel R., Deckers M. (2018). Mitochondrial dysfunction and its role in tissue-specific cellular stress. Cell Stress.

[28] Boengler K., Kosiol M., Mayr M., Schulz R., Rohrbach S. (2017). Mitochondria and ageing: Role in heart, skeletal muscle and adipose tissue. J. Cachexia Sarcopenia Muscle.

[29] Patti M.E., Butte A.J., Crunkhorn S., Cusi K., Berria R., Kashyap S., Miyazaki Y., Kohane I., Costello M., Saccone R. (2003). Coordinated reduction of genes of oxidative metabolism in humans with insulin resistance and diabetes: Potential role of PGC1 and NRF1. Proc. Natl. Acad. Sci. USA.

[30] Mogensen M., Sahlin K., Fernstrom M., Glintborg D., Vind B.F., Beck-Nielsen H., Hojlund K. (2007). Mitochondrial respiration is decreased in skeletal muscle of patients with type 2 diabetes. Diabetes.

[31] Porcu S., Lapolla A., Biasutto L., Zoratti M., Piarulli F., Eliana G., Basso D., Roverso M., Seraglia R. (2014). A preliminary fastview of mitochondrial protein profile from healthy and type 2 diabetic subjects. Eur. J. Mass Spectrom.

[32] Sreekumar R., Halvatsiotis P., Schimke J.C., Nair K.S. (2002). Gene expression profile in skeletal muscle of type 2 diabetes and the effect of insulin treatment. Diabetes.

[33] Bonnard C., Durand A., Peyrol S., Chanseaume E., Chauvin M.A., Morio B., Vidal H., Rieusset J. (2008). Mitochondrial dysfunction results from oxidative stress in the skeletal muscle of diet-induced insulin-resistant mice. J. Clin. Investig..

[34] Pinti M.V., Fink G.K., Hathaway Q.A., Durr A.J., Kunovac A., Hollander J.M. (2019). Mitochondrial dysfunction in type 2 diabetes mellitus: An organ-based analysis. Am. J. Physiol. Endocrinol. Metab..

[35] Bhatti J.S., Bhatti G.K., Reddy P.H. (2017). Mitochondrial dysfunction and oxidative stress in metabolic disorders—A step towards mitochondria based therapeutic strategies. Biochim. Biophys. Acta. Mol. Basis. Dis..

[36] Karabatsiakis A., Bock C., Salinas-Manrique J., Kolassa S., Calzia E., Dietrich D.E., Kolassa I.T. (2014). Mitochondrial respiration in peripheral blood mononuclear cells correlates with depressive subsymptoms and severity of major depression. Transl. Psychiatry.

[37] Tyrrell D.J., Bharadwaj M.S., Jorgensen M.J., Register T.C., Shively C., Andrews R.N., Neth B., Keene C.D., Mintz A., Craft S. (2017). Blood-Based Bioenergetic Profiling Reflects Differences in Brain Bioenergetics and Metabolism. Oxid. Med. Cell Longev.

[38] Kramer P.A., Ravi S., Chacko B., Johnson M.S., Darley-Usmar V.M. (2014). A review of the mitochondrial and glycolytic metabolism in human platelets and leukocytes: Implications for their use as bioenergetic biomarkers. Redox Biol..

[39] Thon J.N., Italiano J.E. (2012). Platelets: Production, morphology and ultrastructure. Handb. Exp. Pharmacol..

[40] Hoppel F., Garcia-Souza L.F., Kantner-Rumplmair W., Burtscher M., Gnaiger E., Pesta D., Calabria E. (2021). Human Platelet Mitochondrial Function Reflects Systemic Mitochondrial Alterations: A Protocol for Application in Field Studies. Cells.

[41] Mossberg K., Olausson J., Fryk E., Jern S., Jansson P.A., Brogren H. (2022). The role of the platelet pool of Plasminogen Activator Inhibitor-1 in well-controlled type 2 diabetes patients. PLoS ONE.

[42] Tschoepe D., Roesen P., Esser J., Schwippert B., Nieuwenhuis H.K., Kehrel B., Gries F.A. (1991). Large platelets circulate in an activated state in diabetes mellitus. Semin. Thromb. Hemost.

[43] Sumbalova Z.D.S., Hiller E., Chang S., Garcia-Souza L., Calabria E., Volani C., Krumschnabel G., Gneiger E. (2018). O2k-Protocols: Isolation of peripheral blood mononuclear cells and platelets from human blood for HRR. MiPNet.

[44] Braganza A., Annarapu G.K., Shiva S. (2020). Blood-based bioenergetics: An emerging translational and clinical tool. Mol. Aspects Med..

[45] Widlansky M.E., Wang J., Shenouda S.M., Hagen T.M., Smith A.R., Kizhakekuttu T.J., Kluge M.A., Weihrauch D., Gutterman D.D., Vita J.A. (2010). Altered mitochondrial membrane potential, mass, and morphology in the mononuclear cells of humans with type 2 diabetes. Transl. Res..

[46] Hartman M.L., Shirihai O.S., Holbrook M., Xu G., Kocherla M., Shah A., Fetterman J.L., Kluge M.A., Frame A.A., Hamburg N.M. (2014). Relation of mitochondrial oxygen consumption in peripheral blood mononuclear cells to vascular function in type 2 diabetes mellitus. Vasc. Med..

[47] American Diabetes A. (2015). (2) Classification and diagnosis of diabetes. Diabetes Care.

[48] Wells J.C., Cole T.J., Steam A.S. (2002). Adjustment of fat-free mass and fat mass for height in children aged 8 y. Int. J. Obes. Relat. Metab. Disord.

[49] Gunther C.M., Burger A., Rickert M., Crispin A., Schulz C.U. (2008). Grip strength in healthy caucasian adults: Reference values. J. Hand Surg. Am..

[50] Jimenez-Pavon D., Ortega F.B., Valtuena J., Castro-Pinero J., Gomez-Martinez S., Zaccaria M., Gottrand F., Molnar D., Sjostrom M., Gonzalez-Gross M. (2012). Muscular strength and markers of insulin resistance in European adolescents: The HELENA Study. Eur. J. Appl. Physiol..

[51] Abdul-Hameed U., Rangra P., Shareef M.Y., Hussain M.E. (2012). Reliability of 1-repetition maximum estimation for upper and lower body muscular strength measurement in untrained middle aged type 2 diabetic patients. Asian J. Sports Med..

[52] Brzycki M. (1993). Strength Testing—Predicting a One-Rep Max from Reps-to-Fatigue. J. Phys. Educ. Recreat. Danc..

[53] Bruseghini P., Calabria E., Tam E., Milanese C., Oliboni E., Pezzato A., Pogliaghi S., Salvagno G.L., Schena F., Mucelli R.P. (2015). Effects of eight weeks of aerobic interval training and of isoinertial resistance training on risk factors of cardiometabolic diseases and exercise capacity in healthy elderly subjects. Oncotarget.

[54] Simundic A.M., Bolenius K., Cadamuro J., Church S., Cornes M.P., van Dongen-Lases E.C., Eker P., Erdeljanovic T., Grankvist K., Guimaraes J.T. (2018). Joint EFLM-COLABIOCLI Recommendation for venous blood sampling. Clin. Chem. Lab. Med..

[55] Venturelli M., Ruzzante F., Villa F., Rudi D., Tarperi C., Milanese C., Cavedon V., Fonte C., Picelli A., Smania N. (2020). Response: Commentary: Neuromuscular and Muscle Metabolic Functions in MELAS Before and After Resistance Training: A Case Study. Front Physiol..

[56] Doerrier C., Garcia-Souza L.F., Krumschnabel G., Wohlfarter Y., Meszaros A.T., Gnaiger E. (2018). High-Resolution FluoRespirometry and OXPHOS Protocols for Human Cells, Permeabilized Fibers from Small Biopsies of Muscle, and Isolated Mitochondria. Methods Mol. Biol..

[57] Faul F., Erdfelder E., Lang A.G., Buchner A. (2007). G*Power 3: A flexible statistical power analysis program for the social, behavioral, and biomedical sciences. Behav. Res. Methods.

[58] Sivitz W.I., Yorek M.A. (2010). Mitochondrial dysfunction in diabetes: From molecular mechanisms to functional significance and therapeutic opportunities. Antioxid Redox Signal.

[59] Mootha V.K., Lindgren C.M., Eriksson K.F., Subramanian A., Sihag S., Lehar J., Puigserver P., Carlsson E., Ridderstrale M., Laurila E. (2003). PGC-1alpha-responsive genes involved in oxidative phosphorylation are coordinately downregulated in human diabetes. Nat. Genet..

[60] Nisoli E., Carruba M.O. (2006). Nitric oxide and mitochondrial biogenesis. J. Cell Sci..

[61] Goodpaster B.H. (2013). Mitochondrial deficiency is associated with insulin resistance. Diabetes.

[62] Holloszy J.O. (2013). "Deficiency" of mitochondria in muscle does not cause insulin resistance. Diabetes.

[63] Kelley D.E., He J., Menshikova E.V., Ritov V.B. (2002). Dysfunction of mitochondria in human skeletal muscle in type 2 diabetes. Diabetes.

[64] Petersen K.F., Dufour S., Befroy D., Garcia R., Shulman G.I. (2004). Impaired mitochondrial activity in the insulin-resistant offspring of patients with type 2 diabetes. N. Engl. J. Med..

[65] Befroy D.E., Petersen K.F., Dufour S., Mason G.F., de Graaf R.A., Rothman D.L., Shulman G.I. (2007). Impaired mitochondrial substrate oxidation in muscle of insulin-resistant offspring of type 2 diabetic patients. Diabetes.

[66] Ritov V.B., Menshikova E.V., He J., Ferrell R.E., Goodpaster B.H., Kelley D.E. (2005). Deficiency of subsarcolemmal mitochondria in obesity and type 2 diabetes. Diabetes.

[67] Ritov V.B., Menshikova E.V., Azuma K., Wood R., Toledo F.G., Goodpaster B.H., Ruderman N.B., Kelley D.E. (2010). Deficiency of electron transport chain in human skeletal muscle mitochondria in type 2 diabetes mellitus and obesity. Am. J. Physiol. Endocrinol. Metab..

[68] Phielix E., Schrauwen-Hinderling V.B., Mensink M., Lenaers E., Meex R., Hoeks J., Kooi M.E., Moonen-Kornips E., Sels J.P., Hesselink M.K. (2008). Lower intrinsic ADP-stimulated mitochondrial respiration underlies in vivo mitochondrial dysfunction in muscle of male type 2 diabetic patients. Diabetes.

[69] Chanseaume E., Barquissau V., Salles J., Aucouturier J., Patrac V., Giraudet C., Gryson C., Duche P., Boirie Y., Chardigny J.M. (2010). Muscle mitochondrial oxidative phosphorylation activity, but not content, is altered with abdominal obesity in sedentary men: Synergism with changes in insulin sensitivity. J. Clin. Endocrinol. Metab..

[70] Lovejoy J.C., Champagne C.M., de Jonge L., Xie H., Smith S.R. (2008). Increased visceral fat and decreased energy expenditure during the menopausal transition. Int. J. Obes..

[71] Neeland I.J., Turer A.T., Ayers C.R., Powell-Wiley T.M., Vega G.L., Farzaneh-Far R., Grundy S.M., Khera A., McGuire D.K., de Lemos J.A. (2012). Dysfunctional adiposity and the risk of prediabetes and type 2 diabetes in obese adults. JAMA.

[72] Smith U. (2015). Abdominal obesity: A marker of ectopic fat accumulation. J. Clin. Investig..

[73] Tyrrell D.J., Bharadwaj M.S., Van Horn C.G., Marsh A.P., Nicklas B.J., Molina A.J. (2015). Blood-cell bioenergetics are associated with physical function and inflammation in overweight/obese older adults. Exp. Gerontol..

[74] Kunz H.E., Hart C.R., Gries K.J., Parvizi M., Laurenti M., Dalla Man C., Moore N., Zhang X., Ryan Z., Polley E.C. (2021). Adipose tissue macrophage populations and inflammation are associated with systemic inflammation and insulin resistance in obesity. Am. J. Physiol. Endocrinol. Metab..

[75] Park S.W., Goodpaster B.H., Strotmeyer E.S., Kuller L.H., Broudeau R., Kammerer C., de Rekeneire N., Harris T.B., Schwartz A.V., Tylavsky F.A. (2007). Accelerated loss of skeletal muscle strength in older adults with type 2 diabetes: The health, aging, and body composition study. Diabetes Care.

[76] Mesinovic J., Zengin A., De Courten B., Ebeling P.R., Scott D. (2019). Sarcopenia and type 2 diabetes mellitus: A bidirectional relationship. Diabetes Metab. Syndr. Obe.s.

[77] Scott D., de Courten B., Ebeling P.R. (2016). Sarcopenia: A potential cause and consequence of type 2 diabetes in Australia’s ageing population?. Med. J. Aust..

[78] Tiainen K., Raitanen J., Strandberg T., Koskinen S., Stenholm S. (2022). Type 2 Diabetes as a Predictor of Muscle Strength Decline over 11 years among Men and Women Aged 55 Years and Older. Gerontology.

[79] Murakami K., Kondo T., Ohtsuka Y., Fujiwara Y., Shimada M., Kawakami Y. (1989). Impairment of glutathione metabolism in erythrocytes from patients with diabetes mellitus. Metabolism.

[80] Venditti P., Di Stefano L., Di Meo S. (2013). Mitochondrial metabolism of reactive oxygen species. Mitochondrion.

[81] Chen G., Chen Z., Hu Y., Huang P. (2011). Inhibition of mitochondrial respiration and rapid depletion of mitochondrial glutathione by beta-phenethyl isothiocyanate: Mechanisms for anti-leukemia activity. Antioxid. Redox. Signal..

[82] Sreekumar P.G., Ferrington D.A., Kannan R. (2021). Glutathione Metabolism and the Novel Role of Mitochondrial GSH in Retinal Degeneration. Antioxidants.

