# Supplementary material for: Type 2 Diabetes Related Mitochondrial Defects in Peripheral Mononucleated Blood Cells from Overweight Postmenopausal Women
Source: Biomedicines. 2023 Jan 3;11(1):121. doi: 10.3390/biomedicines11010121 (PMC9855941; doi:10.3390/biomedicines11010121)

Supplementary Table S1 – Hematological and hematochemical parameters obtained from blood samples of the participants. Data are reported as mean ± st.dev; (CTRL N=9; T2D N=9).

**Description**	**CTRL**	**T2D**	P value (Unpaired Student's t-test P < 0.05)
N	9	9	
RBC (10^9/L)	4.60 ± 0.3	4.34 ±0.3	0.09
WBC (10^9/L)	5.24 ± 1.12	6.42 ± 1.35	0.22
Lymphocytes (%)	39.70 ± 8.03	37.24 ± 9.32	0.57
Monocytes (%)	8.59 ± 2.52	8.52 ± 1.11	0.93
Neutrophyles (%)	48.88± 8.69	50.80 ± 8.17	0.81
Eosinophyles (%)	2.30 ± 1.13	2.62 ± 1.06	0.55
Basophyles (%)	0.46 ± 0.24	0.82 ± 0.39	* 0.03
PLTs	255.50± 33.90	223.22 ± 24.92	0.07

Supplementary Figure S1 – Graphical representation of the types of cells present in purified PBMCs samples. a- Percentage of lymphocytes (LYMPH) and monocytes (MONO) in PBMCs samples from CTRL and T2D groups. b, c- residual contamination form neutrophiles (NEU) and platelets (PLTs) is reported as NEU/PBMCs and PLTs/PBMCs ratios. . Data are reported as mean ± st.dev; (CTRL N=9; T2D N=9)

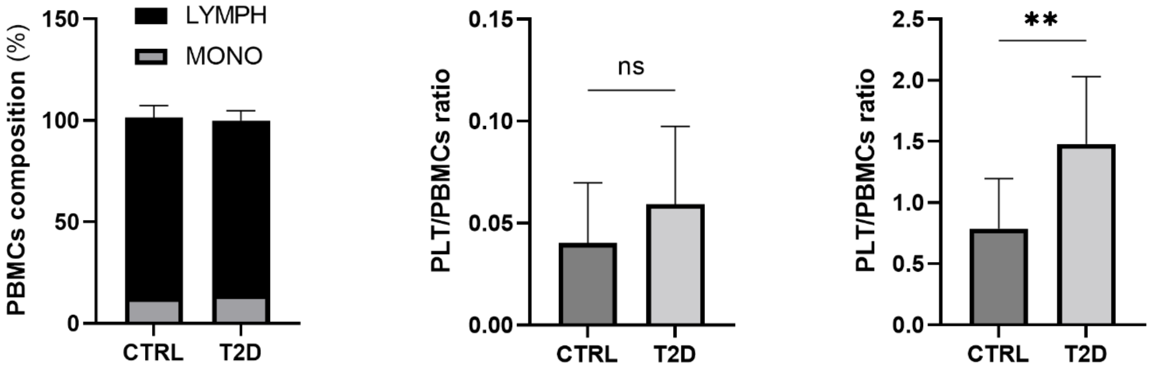

Supplementary Figure S2 - Tree diagram of hierarchical grouping considering markers for all the participants.

Hierarchical clustering diagram describes the association between all the covariates included in the analysis. The centroid method of hierarchical cluster analysis based on the linkage distances was applied to determine relatively homogeneous groups. From the dendrogram, we can observe the distance between subjects, and how the different clusters allocate the participants from the two groups.

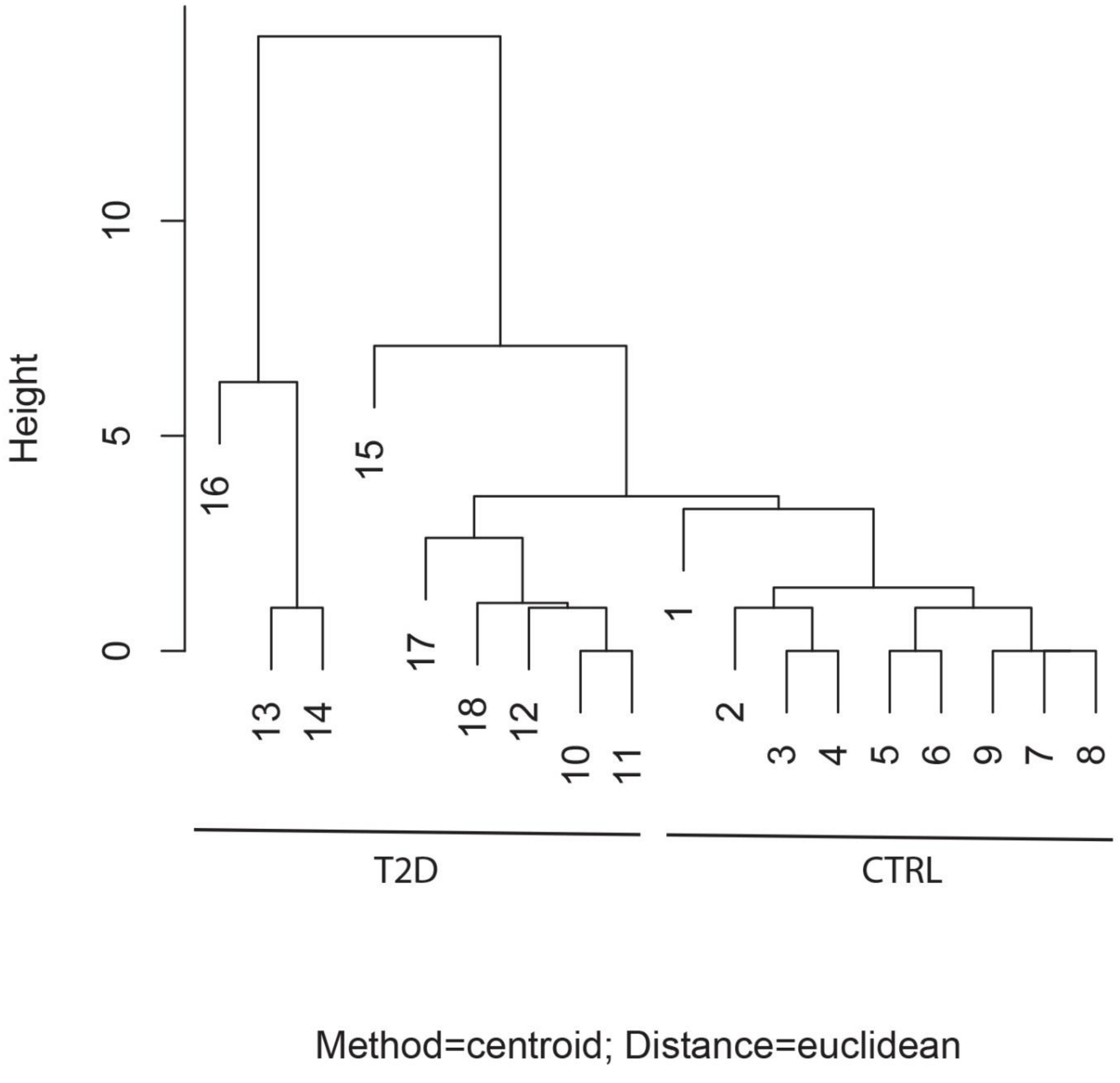

Supplement: Supplementary file 1 [file biomedicines-11-00121-s001.zip › biomedicines-2035536-supplementary.pdf]
